# Mitochondrial dysfunction and Alzheimer’s disease: pathogenesis of mitochondrial transfer

**DOI:** 10.3389/fnagi.2024.1517965

**Published:** 2024-12-17

**Authors:** Yun Wei, Xinlei Du, Hongling Guo, Jingjing Han, Meixia Liu

**Affiliations:** Xiyuan Hospital of China Academy of Chinese Medical Sciences, Beijing, China

**Keywords:** Alzheimer’s disease, mitochondrial transfer, mitochondrial dysfunction, neuroprotection, AD treatment

## Abstract

In recent years, mitochondrial transfer has emerged as a universal phenomenon intertwined with various systemic physiological and pathological processes. Alzheimer’s disease (AD) is a multifactorial disease, with mitochondrial dysfunction at its core. Although numerous studies have found evidence of mitochondrial transfer in AD models, the precise mechanisms remain unclear. Recent studies have revealed the dynamic transfer of mitochondria in Alzheimer’s disease, not only between nerve cells and glial cells, but also between nerve cells and glial cells. In this review, we explore the pathways and mechanisms of mitochondrial transfer in Alzheimer’s disease and how these transfer activities contribute to disease progression.

## 1 Background

The authors reviewed scientific literature (PubMed, meeting abstracts, and websites) on mitochondrial transfer in AD. Numerous studies have found evidence and key role of mitochondrial transfer in AD models, and relevant publications are cited throughout the manuscript. In this review, the phenomenon of mitochondrial transfer is described in terms of its function under physiological and pathological conditions, including tissue homeostasis, damaged tissue repair, and dementia progression. Then, the mechanism of this process in AD is summarized, such as trigger factors and transfer pathways. In addition, various perspectives are explored to better understand the mysteries of cell-to-cell mitochondrial transport.

## 2 Introduction

In the central nervous system (CNS), neuron function hinges extensively on mitochondrial integrity and activity. As a crucial powerhouse for cellular metabolism and tissue survival, mitochondria often adapt their morphology and position in response to various stressors and energy demands. Alzheimer’s disease (AD) represents one of the most prevalent forms of dementia worldwide, characterized by an incurable progression that typically results in the patient’s death. Recently, the mitochondrial cascade hypothesis has gained prominence, emphasizing the pivotal role of mitochondrial bioenergetics in AD (Swerdlow, 2018). This hypothesis posits that mitochondrial dysfunction is a preliminary and critical event that aggravates the pathological cascade (Sharma et al., 2021). While the question of whether mitochondrial-related changes contribute to AD’s development or are its consequence remains open, a clear association between mitochondrial dysfunction and AD has been extensively demonstrated.

Mitochondria can be spontaneous transferred intracellularly or intercellularly both *in vivo* and *in vitro*, by a process known as “mitochondrial transfer” (Chen et al., 2022). This phenomenon involves the incorporation of mitochondrial genes or mitochondria into recipient cells. Intercellular mitochondria transfer was initially documented by Koyanagi et al. (2005), and more recent research has underscored the significance in the CNS, particularly in the context of neurodegenerative diseases such as AD (Nitzan et al., 2019). Studies have reported decreased velocity and total travel distance of mitochondria in AD neuronal processes (Yu et al., 2016). However, the physiological nature and specific mechanisms underlying mitochondrial transfer remain unclear.

The study of mitochondrial transfer between and within cells has attracted increasing attention owing to the core functions of mitochondria in AD. This review provides an overview of mitochondrial dysfunction’s role in AD pathology, elucidates the primary mechanisms governing mitochondrial transfer among and within cells, and underscores the significance of this phenomenon. In doing so, it introduces fresh perspectives and potential targets for advancing AD treatment strategies.

### 2.1 Origin of mitochondrial changes in AD

Mitochondria are dynamic organelles that continually adapt their structure and metabolism to meet cellular demands. Their role is pivotal in preserving neurovascular function within the CNS, whether in physiological or pathophysiological states (Rutkai et al., 2019). In AD, mitochondrial dysfunction manifests through various facets, including reduced mitochondrial size (Wang et al., 2005), increased mitochondrial DNA (mtDNA) damage, decreased synaptic adenosine triphosphate (ATP) levels, impaired mtDNA expression, reduced mtDNA copies, increased oxidative damage, reduced mitochondrial axonal transport, and glucose hypometabolism (Sang et al., 2018). However, the source of these mitochondrial changes in AD remains debatable.

### 2.2 Amyloid-*β* (Aβ) induces AD mitochondrial dysfunction

Aβ has been implicated in senile plaque formation and neuronal apoptosis, and it plays a crucial role in AD pathogenesis. An increasing body of evidence suggests that Aβ monomers and oligomers can inhibit axonal mitochondrial transport and interrupt the mitochondrial fusion/fission balance. For example, Guo et al. (2013) observed a significant 30–40% reduction in mitochondrial density and movement in axons exposed to 200 nM oligomeric Aβ, all without affecting cell viability. Additionally, studies (Devi et al., 2006) found that Amyloid Precursor Protein (APP) (full length or C-terminal fragment) and Aβ were associated with the mitochondrial membrane in AD human brains brain regions. This suggests that Aβ-induced mitochondrial dysfunction in AD occurs through both direct and indirect mechanisms, with early alterations in axonal mitochondrial transport preceding neuronal death. Conversely, excessive mitochondrial division also leads to Aβ-mediated neuropathological and cognitive decline (Qi et al., 2019). In other words, mitochondrial dysfunction may occur independently of Aβ and may be an upstream contributor to Aβ deposition in AD.

### 2.3 Tau induces AD mitochondrial dysfunction

Apart from Aβ, overexpression and/or hyperphosphorylation of Tau has also been shown to disrupt both the distribution and localization of mitochondria in models and patient of AD. Tau is a hydrophobic protein that stabilizes the neuronal microtubules and regulates axonal transport. In the human AD brain, the distribution of mitochondria in neurites containing tau aggregates is disrupted in an age-dependent manner in the human AD brain (Kopeikina et al., 2011). Pérez et al. (2018) transfected a plasmid with different forms of Tau labeled with green fluorescent protein in Tau gene-knockout mice and found that cells expressing truncated Tau showed significantly lower optic atrophy 1 (OPA1) levels and mitochondrial fragmentation. Moreover, Tau inhibits mitochondrial Ca^2+^ efflux via the mitochondrial Na^+^/Ca^2+^ exchanger, leading to mitochondrial depolarization in response to stimuli inducing Ca^2+^ signaling and cell death. Moreover, high concentrations of Tau protein can affect the dynamic balance between mitochondrial division and fusion, thus interfering with mitochondrial transport. Consequently, mitochondrial dynamics disorders arising from pathological changes due to abnormally expressed Tau protein plays an important role in AD pathogenesis. However, the mechanisms underlying the influence of the Tau protein on mitochondria warrant further exploration.

### 2.4 Aβ-tau interaction induce AD mitochondrial dysfunction

Interestingly, some studies have hypothesized that Aβ and Tau may interact to degrade mitochondrial dysfunction in AD (John and Reddy, 2021). The effects of Aβ species on mitochondrial movement is subject to the presence of Tau, with one study suggesting that Aβ mainly causes a complex IV defect, while Tau mainly affects complex I (Rhein et al., 2009). Another study found that compared with an AD mouse model with a single distinct pathology, the synergistic effects of both pathologies in a 3xTg-AD mouse model occur by impacting oxidative phosphorylation system (OXPHOS) (Kim and Mook-Jung, 2019). At present, knowledge regarding Aβ-tau synergy is still in its infancy, and many unknown factors remain, such as the different pathological causes and manifestations of mitochondrial damage in different models, which need further study.

**Figure 1:**
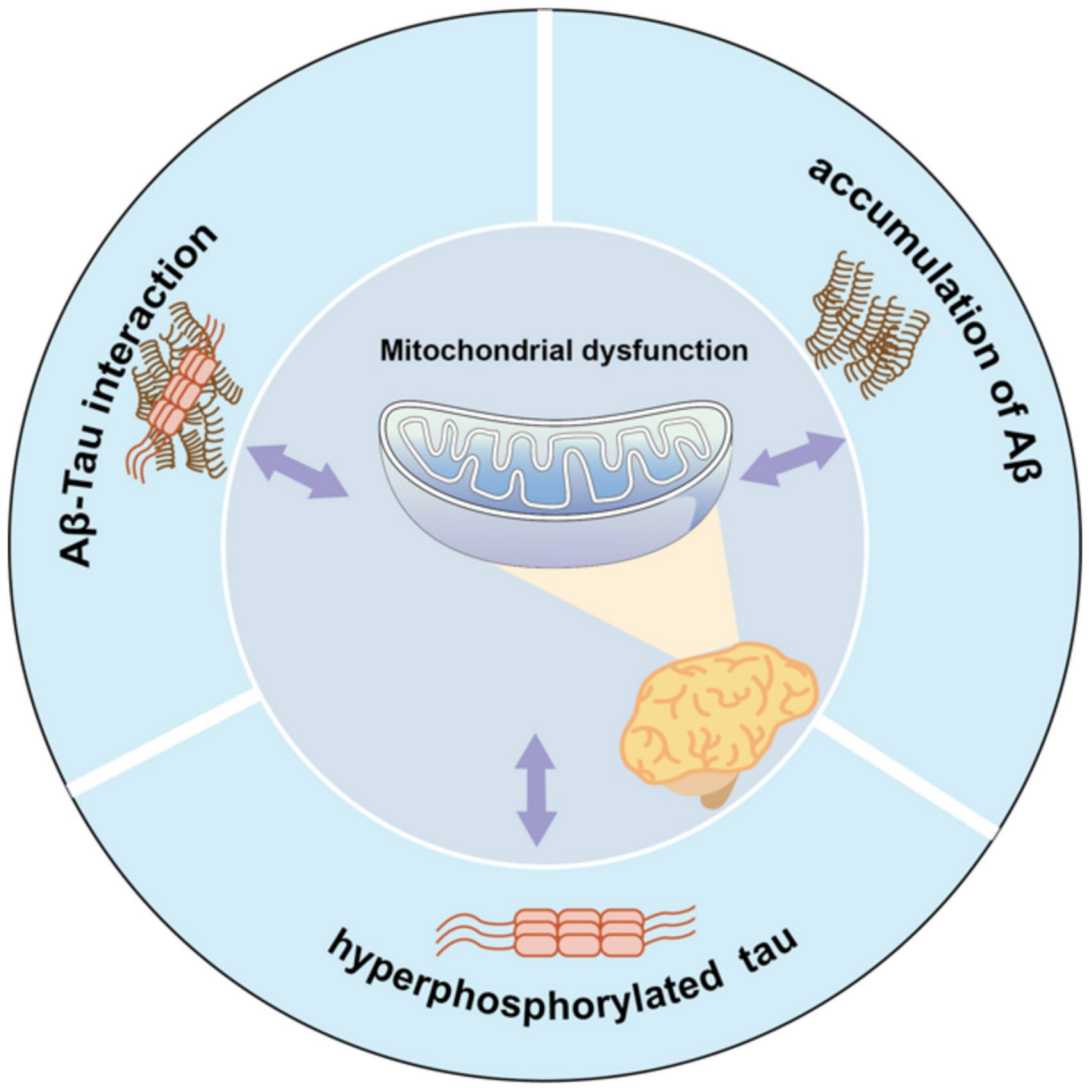
Mitochondrial dysfunction and pathological features form a vicious cycle in AD pathology. Aβ accumulation, Tau hyperphosphorylation, or Aβ-Tau interaction can all contribute to mitochondrial dysfunction, which in turn exacerbates abnormalities in all three, which further impair mitochondrial function.

Furthermore, abnormal secretion of apolipoprotein E4 (Reiman et al., 2004) and oxidative stress (Swomley and Butterfield, 2015) may also be related to a rise in ROS/RNS levels, leading to oxidation of mitochondrial proteins, lipids, and DNA. Thus, the relationship among the multiple mechanisms of mitochondrial dysfunction in AD is complex, with mechanisms potentially influencing each other (Figure 1). Excessive Aβ accumulation, abnormal forms of Tau protein, and Aβ-Tau interaction can individually or collectively disrupt mitochondrial axon transport, affecting nutrient delivery and synaptic information exchange. These disruptions may represent the primary contributors to learning and memory disorders.

Mitochondrial dysfunction and its associated pathological features form a vicious cycle in AD. Aβ accumulation, Tau hyperphosphorylation, or Aβ-Tau interaction can each instigate mitochondrial dysfunction. Consequently, these abnormalities collectively exacerbate the condition of all three factors, further impairing mitochondrial function.

## 3 Structural basis of mitochondrial transfer

Mitochondria are highly dynamic organelles that can move within and between subcellular compartments closely linked to neuroplasticity, such as synaptic terminals, dendrites, cell bodies, and axons, resulting in changes in their form, length, size and number, which in turn affect the energy, vitality, and even cell death. Researchers use cybrid cells with incorporated platelet mitochondria from AD or age-matched non-AD human subjects into mtDNA-depleted neuronal cells (SH-SY5Y) to prepare AD or non-AD hybrid cells, and found in non-AD cybrid cells; for instance, mitochondria are shorter and scattered along the differentiated neuronal processes, whereas in AD cells, the mitochondria are concentrated within the shortened processes (Yu et al., 2016). Du et al. (2021) observed a fission-pattern with fragmented and scattered mitochondria in AD cells as opposed to jointed and elongated mitochondria distributed in the differentiated normal non-AD cells. Consequently, the versatile and ever-changing nature of mitochondria underscores their dynamic nature, implying the possible existence of mitochondrial transfer in both *in vivo* and *in vitro* experiments. While the link between the phenotype and mitochondrial function is not always observed, under many conditions, actively respirating mitochondria are long and filamentous, while short, round fragmented mitochondria are often associated with pathological conditions.

The level of neuronal activity stands as a direct determinant of mitochondrial morphology and abundance during mitochondrial intracellular transfer. Neurons control the mobility, distribution, and clearance of mitochondria to maintain their energy balance and prevent oxidative stress. In mature neurons (Sheng, 2014), approximately 20–30% of axonal mitochondria exhibit motility, while roughly 15% undergo brief pausing or docking at synapses. Approximately 14% of motile mitochondria dynamically pass through presynaptic terminals, and their transport is modulated in response to physiological signals. This mobility allows mitochondria to adapt their metabolic state and function in response to changes in the external environment over time. Meanwhile, fission may be necessary for intercellular mitochondrial transfer (Zampieri et al., 2021) because smaller, non-elongated mitochondria are mor easily internalized by the recipient cell. Other researchers found that (Geng et al., 2023) astrocytes can deliver 300–1,100 nm mitochondrial particles. Moreover, transferred mitochondria lack a membrane potential, because dysfunctional mitochondria that lack a membrane potential are normally degraded or repaired by fusion with healthy mitochondrial networks (Phinney et al., 2015). Different conclusions may be related to different cell types, cell viability, and different cellular environments (Table 1).

**Table 1:** Mitochondrial morphological changes in different Alzheimer’s disease models.

Sources	Mitochondrial morphology	Distribution	References
Normal	Short round-shaped or elongated organelles, with a major axis reaching 5 μm in length	Widely distributed in the cytoplasmic matrix	Cereghetti and Scorrano (2006)
Fibroblast cell lines from sAD patients	sAD and healthy cells exhibit similar mitochondrial patterns under resting conditions with most cells showing a filamentous and intermediate morphology	Accumulate in perinuclear areas	Martín-Maestro et al. (2017)
Fibroblast cell lines from sAD patients	significantly longer (>30 μm) compared to the short and rounded mitochondria of age-matched normal human fibroblasts	Mainly concentrated in the perinuclear area and were sparse in remote and peripheral regions of the cells	Wang et al. (2008)
APPwt and APPswe M17 cells	Exhibit a fragmented structure	Accumulate around the perinuclear area	Wang et al. (2008)
OXYS rats, a model of the sporadic AD	Axons exhibite segments with a spindle-like shape alternating with narrower segments measuring approximately 1 μm in length. Moreover, no significant differences in the total quantity of mitochondria, including ovoid, elongated, and MOAS forms, are found within the cortex axons of both Wistar and OXYS rats, and this quantity remained stable with aging.	–	Swomley and Butterfield (2015)
Mitochondria in the CA1 hippocampal region from APP mice, PS1 mice, Tau mice, APP/PS1 mice, 3xTgAD mice and AD patients	MOAS mitochondria exhibit ovoid shapes (0.3 * 0.5 μm in diameter) with teardrop profiles featuring tubular membrane extension(s) at one or both ends. Additionally, there were teardrop shaped mitochondria (0.5 μm in diameter) connected by a thin double membrane extending up to 5 μm in length.	–	Zhang et al. (2016)
H4PS1Δexon9 cell line upon PS1 mutant induction	Increase in fragmented mitochondria, which were shortened, punctate, and spherical	Aggregated mitochondria in the cytosol	Han et al. (2021)
sAD patients	Lower percentage of normal mitochondria as well as relatively high percentage of mitochondria with broken cristae	–	Hirai et al. (2001)

sAD, sporadic Alzheimer’s disease; APP, amyloid precursor protein; MOAS, mitochondria-on-a-string.

## 4 Directionality of mitochondrial transfer

Generally, intra- and inter-cellular mitochondrial transfer displays varying degrees of directional, saltatory, and bidirectional movement, and it is determined by several factors. Ruthel and Hollenbeck (2003) reported that intracellular mitochondrial transfer results from specific directed motility rather than bulk flow in the axoplasm or organelles. Other studies have demonstrated that the directionality of intracellular mitochondrial transfer is associated with cytoskeletal microfilaments in microvesicles of connected tubular structures (Ahmad et al., 2014; Yasuda et al., 2011). Additionally, Aβ protein concentration is also thought to affect the direction of intracellular mitochondrial transfer. Furthermore, low Aβ levels can impair anterograde transport while enhancing retrograde axonal mitochondrial movement by disrupting microtubule stability via decreased *α*-tubulin acetylation (Du et al., 2010; Wang et al., 2015). Moreover, hippocampal neurons treated with Aβ-derived diffusible ligands develop impaired axonal transport of mitochondria in both anterograde and retrograde directions (Wang et al., 2009).

Concerning the direction of intercellular mitochondrial transfer, Plotnikov et al. (2008) established that intercellular cytoplasmic transfer occurs in a nondirectional manner. Of course, some scholars believe that the direction of transfer is related to the cell state. In a typical intercellular mitochondrial transfer, recipient cells are characterized by elevated OXPHO needs and severely compromised mitochondrial functional (Marlein et al., 2019; Moschoi et al., 2016), while donor cells with proficient mitochondrial function are appropriately activated (Marlein et al., 2018). Although bidirectional mitochondrial transfer has occasionally been observed, the precise direction and mechanism of such transfer remain unclear.

## 5 Function of mitochondrial transfer in AD

In the brain, bidirectional mitochondrial transfer within neurons, between glia and other glial cells, and between glia and neurons has been observed; the delivered mitochondria are internalized and degraded (Davis et al., 2014) or even rescue signal transport (Liu et al., 2021). Although extracellular damaged mitochondria are injurious, transfer of functional mitochondria is protective. The ratio between damaged mitochondria and functional mitochondria in the extracellular milieu governs the outcome in neurons (Joshi et al., 2019). Mitochondrial transfer is a ubiquitous phenomenon associated with various physiological and pathological processes.

Mitochondrial dysfunction is an early and prominent feature of AD (Wang et al., 2014). The intracellular and intercellular transfer of mitochondria is involved in the mitochondrial quality control process and jointly maintains the mitochondrial homeostasis of local tissues or the whole body, which provides a promising therapeutic target for AD. Intracellular mitochondrial transfer, especially bidirectional mitochondrial transport within astrocytes, microglia, and neurons (Hayakawa et al., 2016; Lippert and Borlongan, 2019), is capable of eliminating damaged mitochondria or restoring healthy mitochondria and is necessary to meet the dynamic energy requirements of different regions of neurons. At the same time, intercellular mitochondrial transfer has also proved to be a biological event that cannot be ignored in the CNS. This transcellular transfer of mitochondria promotes the integration of the transferred mitochondria into the endogenous network of the recipient cell, participates in inflammatory or oxidative stress processes, regulates neuronal [Ca^2+^_i_] levels and health (Joshi et al., 2019; Padilla-Sánchez et al., 2020; English et al., 2020; Lampinen et al., 2022; Sun et al., 2012; Zhang et al., 2020), contributes to changes in the bioenergy profile of the recipient cell and other functional properties, and exerts important implications in AD. One study verified that instead of degrading dysfunctional mitochondria through mitophagy, neurons in an AD mouse model transfer dysfunctional mitochondria to neighboring astrocytes (Lampinen et al., 2022), which contributes to neuronal mitochondrial homeostasis. English et al. (2020) have found that cisplatin-treated neurons acquire 35% more mitochondria than healthy neurons, and receptor neurons exhibit improved survival, mitochondrial membrane potential, and calcium homeostasis, in a Miro1-dependent manner. However, while the advantages of mitochondrial transfer are evident, the underlying process remains unclear. Typically, cellular stress or injury is a prerequisite for organelle transfer to occur. When mitochondrial function is relatively intact, mitochondrial transfer is rare (Islam et al., 2012), and it is triggered only when mitochondrial function is almost completely missing (such as when mtDNA is missing or when cells are exposed to mitochondrial inhibitors) (Berridge et al., 2016). Hence, the precise significance of mitochondrial exchange in the maintenance of tissue homeostasis remains unclear.

Here, we summarize the functions of intercellular mitochondrial transfer in AD models (Table 2).

**Table 2:** Function of the intercellular mitochondrial transfer in AD models.

Donor	Acceptor	Induction factor	Components	Transfer outcomes	Routes	Model	References
Microglia	Astrocytes	LPS/nigericin-treated	Fragmented mitochondria	Neuroprotective	EVs, exocytosis	5XFAD mice	Joshi et al. (2019)
Neurons	Astrocytes	H_2_O_2_ treatment	Mitochondria	Elevation of levels of ROS in astrocytes	TNT	5xFAD mice	Lampinen et al. (2022)
Astrocytes	Astrocytes and neurons	H2O2 treatment	Mitochondria	–	TNT	APP/PS1 mouse	Sun et al. (2012)
Human umbilical cord derived MSCs (hucMSCs)	OA-treated SH-SY5Y cells	–	Mitochondria	attenuate oxidative stress	EVs	Okadaic acid (OA)-treated SH-SY5Y cells	Zhang et al. (2020)

α-Syn, α-synuclein; EV, extracellular vesicle; H_2_O_2_, hydrogen peroxide; IV, intravenous; MVs, microvesicles; ROS, reactive oxygen species; TNT, tunneling nanotube; UV, ultraviolet.

## 6 Mechanisms of mitochondrial transfer in AD

In recent years, the transportation of mitochondria within distinct cellular regions or between cells has garnered significance as a pivotal regulator of cellular health, but the mechanisms of intracellular and intercellular transfer may differ. Mitochondrial transfer through these different structures can lead to different functional outcomes for the recipient cells, i.e., functional mitochondrial acquisition, immune activation, or trans-mitophagy (Shanmughapriya et al., 2020). Therefore, a clear understanding of the mechanisms that mediate mitochondrial transfer will shed light on how this process is regulated and can be exploited for therapeutic purposes.

### 6.1 Intracellular mitochondrial transfer

**Figure 2:**
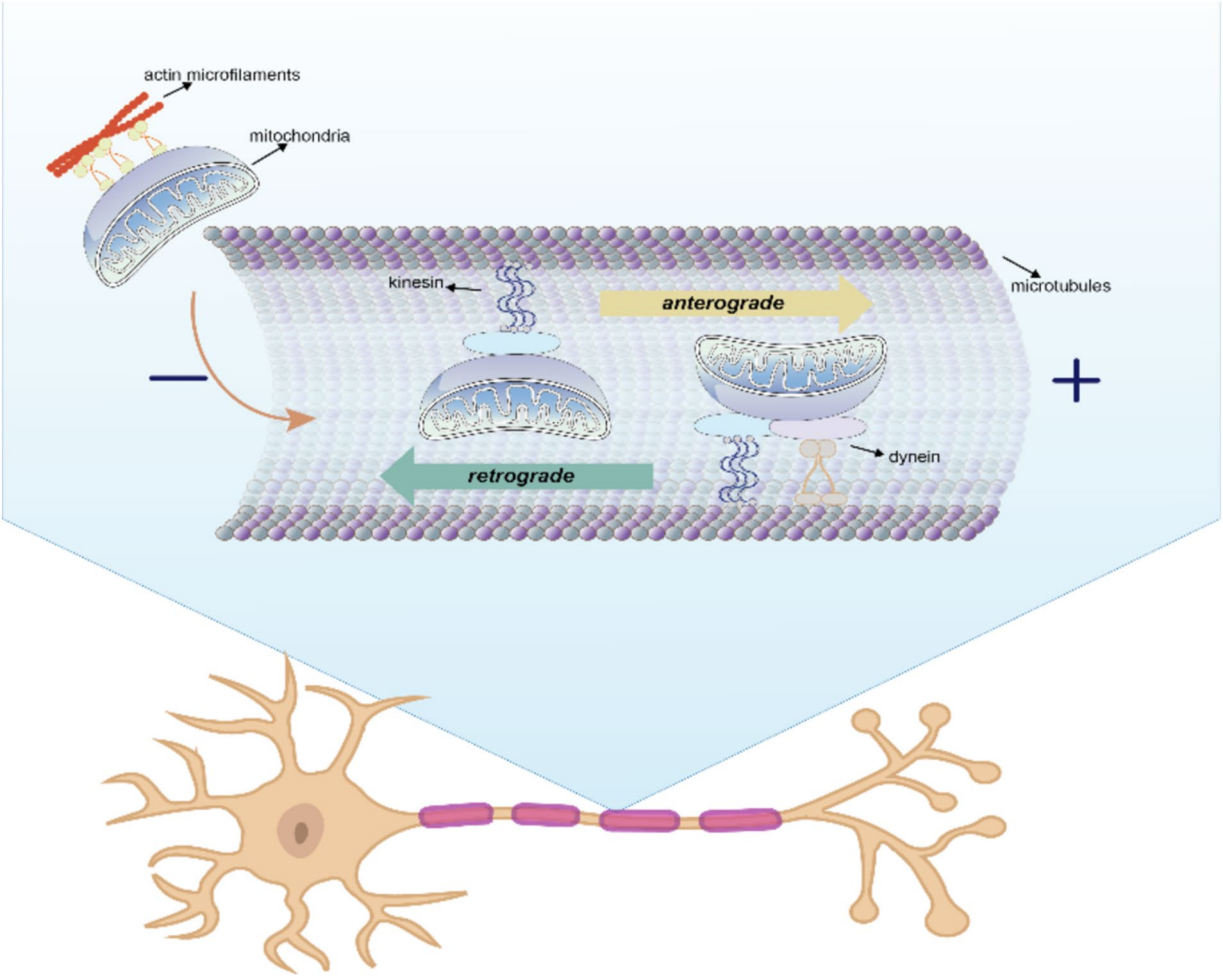
Anterograde and retrograde directions of intracellular mitochondrial movement. In this process, kinesin is responsible for moving mitochondria in an anterograde direction toward the nerve terminal, whereas kinesin and dynein move mitochondria in a retrograde direction toward the soma.

To meet different physiological needs, mitochondria are dynamically transported to specific cellular locations where they perform their functions. The intracellular transfer of mitochondria was first observed in neurons with long axons (Weiss and Mayr, 1971). The state of axon growth determines the proportion of mitochondria that are in motion or at rest: in the region of the active growth cone, the transition of mitochondria from motion to rest is reversed when axon growth is blocked. In addition, neuronal activity increases the mitochondrial transport in synapses. Within these neurons, mitochondria traverse from the cell body to provide energy for long-distance processing and return to the cell body afterward, exhibiting both anterograde (from the nucleus to mitochondria) and retrograde (from mitochondria to nucleus) motility as the classical mechanism of mitochondrial movement. The differences between the two types of intracellular mitochondrial motion patterns are shown in Figure 2 and Table 3.

**Table 3:** Differences between the two patterns of intracellular mitochondrial movement.

	Route	Motor proteins involved	Possible causes (Cheng et al., 2018)	Speed of mitochondrial movement (Niescier et al., 2016)	Significance	Correlation between the direction of transport and mitochondrial inner-membrane potential (Miller and Sheetz, 2004)	Changes in AD (Calkins and Reddy, 2011)
Anterograde movement	From the cell body to the axon	Adaptor proteins such as syntabulin, MIRO and Milton are associated with motor proteins of the kinesin-1 and kinesin-3 family	Oxygen deprivation,	Faster than retrogradely moving mitochondria, and the velocity of anterograde movement varies along the region of the axon traveled	Deliver metabolic energy to synaptic terminals	90% of mitochondria with high membrane potential move anterogradely	Decrease
Retrograde movement	From axon to cell body	Dynein and dynactin proteins	Calcium ion and ROS	Relatively slow	Repair or remove damaged mitochondria in axons	80% of those with low potential move retrogradely	Increase

Kinesin is responsible for moving mitochondria in an anterograde direction toward the nerve terminal, whereas kinesin and dynein move mitochondria in a retrograde direction toward the soma (Seiler et al., 1999). Microtubules serve as rails for the long-range transport of mitochondria, whereas actin microfilaments in dendritic spines, growth cones, and synaptic buds are mainly used for docking and short-range movements (Hirokawa and Takemura, 2005). Intracellular locomotion of mitochondria is based on the interaction of motor and adapter proteins with the microtubule cytoskeleton to transport mitochondria across the neuron. An APP mouse model demonstrated impaired anterograde movement, while in presenilin-1 (PSEN1) and APP/PSEN1 mouse models (Trushina et al., 2012), both retrograde and anterograde transport were compromised. These findings strongly indicate the presence of intracellular mitochondrial transfer in AD.

### 6.2 Intercellular mitochondrial transfer

Recently, a novel mechanism for mitochondrial movement, known as transcellular mitochondria transfer, has emerged (Shanmughapriya et al., 2020). In the context of AD, astrocytes play a crucial role in rescuing damaged neurons after cerebral ischemia by providing them with healthy mitochondria (Fiebig et al., 2019). Conversely, neurons release damaged mitochondria, which are then transferred to astrocytes for disposal and recycling. Moreover, the presence of extracellular particles containing mitochondria in conditioned media from rat cortical astrocytes has been confirmed through electron microscopy (Hayakawa et al., 2016). Transcellular mitochondrial transfer involves various structures and processes, including tunneling nanotubes (TNTs), membrane microvesicles (MVs), gap junctions, cell fusion, and mitochondrial expulsion, and it can lead to significant changes in various biological functions.

#### 6.2.1 Tunneling nanotubes (TNTs)

In 2004, Vandendriessche et al. (2020) first detected the movement of organelles between mammalian cells via TNTs. These TNTs consist of the cell membrane, F-actin, myosin, and tubulin. They serve as conduits for bidirectional movement of a wide range of substances, including small molecules, nearly all neurodegenerative proteins (including Tau (Tardivel et al., 2016) and Aβ (Dilna et al., 2021)), organelles, various ions, and even viral particles (Panasiuk et al., 2018). Therefore, the impact of intercellular TNTs is likely determined by both the transferred cargo and the types and states of connected cells.

Among the transported cargoes that have been described, the mitochondrion appears to be the most frequently reported organelle that can be unidirectionally or bidirectionally transferred via TNTs (Driscoll et al., 2022). Accumulating evidence suggests that the intracellular accumulation of damaged mitochondria can induce the biogenesis of TNT-based mitochondrial transfer (Torralba et al., 2016) in AD (Valappil et al., 2022). However, the mechanisms by which TNTs precisely target recipient cells remain elusive. While the transport of mitochondria via TNTs is well defined, the directionality of mitochondrial transfer and other factors essential for initiating and promoting TNT formation in AD are not fully understood. On the contrary, other research argues against this notion, finding that the inhibiting TNTs did not impact mitochondrial transfer, despite the frequent observation of mitochondria within these structures.

#### 6.2.2 Extracellular vesicles (EVs)

EVs are bilayer membranous structures derived from the endosomal system or shed from the plasma membrane, and they serve as crucial carriers for mitochondrial transport. EVs are found in different types based on their origin, size, and molecular composition, including exosomes (30–100 nm in diameter), MVs (100 nm–1 μm in diameter) and apoptotic bodies (1–2 μm), depending on their origin, size, and molecular constitution (van Niel et al., 2018; Sardar Sinha et al., 2018). They contain cellular proteins, nucleic acids, and organelles that can reach all parts of the body through the circulation and other body fluids due to differences in mitochondrial pressure. Evidence indicates that various cell types, such as astrocytes, neurons (Vandendriessche et al., 2020), mesenchymal stem cells (Phinney et al., 2015), epithelial cells, immune cells (Hosseinkhani et al., 2018), and hepatocytes (Cai et al., 2017), release EVs, which play a pivotal role in intercellular communication during numerous pathophysiological processes (Iraci et al., 2016).

Although the mechanisms by which mitochondrial proteins or mtDNA are loaded into EVs remain unknown, mitochondrial components have been detected in EVs. Three different types of extracellular mitochondrial contents within EVs have been reported: free mtDNA, functional mitochondria, and mitochondrial content (Amari and Germain, 2021). MV-mediated mitochondrial transfer enhances macrophage function by improving mitochondrial bioenergetics. Several studies have clearly highlighted the harmful role of EVs in the pathogenesis of AD (Aulston et al., 2019; Winston et al., 2016). One study found that neuronal- and astrocytic-origin-enriched EVs from plasma and human cells were carriers of AD pathogenic proteins (Nogueras-Ortiz et al., 2020). Specifically, small EVs spread mitochondrial particles, mitochondria (Li et al., 2020), and toxic proteins (Sardar Sinha et al., 2018), inducing neuronal loss and contributing to neuroinflammation and AD progression.

#### 6.2.3 Gap junctions

Gap junctions are plasma membrane channels composed of connexins that transfer molecules of up to 1,000 Daltons (Ishikawa et al., 2012), including nutrients, metabolites, second messengers, cations, anions, and whole mitochondria (Norris, 2021) between cells. Norris (2021) found that mitochondria and endosomes are incorporated into double-membrane vesicles, called connexosomes or annular gap junctions, that form as a result of gap junction internalization. This process may facilitate not only the transfer of mitochondria, but also the release of small molecules from the enclosed mitochondria into the receiving cell cytosol.

It is hypothesized that neuronal and glial gap junctions play a role in propagating neuronal damage in AD models (Pechlivanidou et al., 2022). In older 5XFAD mice (Angeli et al., 2020), connectivity of astrocyte-oligodendrocyte gap junctions decreases, and the expression of gap junctions protein connexin 43 (Cx43) appears to shift, favoring astrocyte-astrocyte gap junctions and/or hemichannels, which could impair oligodendrocyte homeostasis and myelination. Recent studies (Berridge et al., 2016) indicate that Cx43, which mediates the connection between mitochondrial particles and the plasma membrane to form channels, is required for mitochondrial transfer. When gap junctions are inhibited, the benefits of respiratory capacity and ATP synthesis rapidly decline (Li et al., 2019).

#### 6.2.4 Mitochondrial extrusion

Mitochondrial extrusion is another possible mechanism by which naked mitochondria or mitochondrial components are transferred from one cell to another. The mechanism of recognition and extrusion of such damaged mitochondrial components seems to involve the protein Parkin (Crewe et al., 2021). Increasing evidence suggests that the ejection of damaged mitochondria plays an important role in maintaining intracellular mitochondrial quality. This process occurs during developmental processes, inflammatory activation (Liu et al., 2021), or in post-mitotic cells (Lyamzaev et al., 2022) when mitochondria become unfit to remain in the cells. For example, HeLa cells extruded fragmented mitochondria for extracellular mitoptosis under reactive oxygen species (ROS) stress (Lyamzaev et al., 2008). Notably, intact actin and tubulin cytoskeletons and mitochondrial fragmentation are prerequisites for mitochondrial extrusion (Nakajima et al., 2008).

Mitochondrial extrusion is a phenomenon that occurs in response to tumor necrosis factor *α*-induced cell death (Nakajima et al., 2008); this process occurs both *in vitro* and *in vivo* and involves the formation of cytoplasmic vacuoles originating in the plasma membrane—these vacuoles engulf fragmented mitochondria and expel them into the extracellular space, contributing to AD induction (König and McBride, 2024). Potential mechanisms include selective packing into mitochondrial-derived vesicles (MDVs) or autophagosomes before ejection into extracellular spaces, or direct transportation via a mitocytosis-like process (Fan et al., 2022). The mechanisms underlying the ejection of damaged mitochondria in AD are not yet fully understood.

### 6.3 Partial or complete cell fusion

Cell fusion is a process in which two or more individual cells fuse their plasma membranes, sharing organelles and cytosolic compounds, while their individual nuclei remain intact. This process can be triggered by injury and inflammation (Aguilar et al., 2013). Cell fusion plays a crucial role in several physiological and pathological processes (Lee and Chen, 2019), including mitochondrial transfer. Acquistapace et al. (2011) co-cultured human adipose stem cells with mouse cardiomyocytes and found that F-actin junctions were formed between the cells, indicating that mitochondria participate in the recovery of cell function through partial cell fusion. Similarly, Spees et al. (2006) demonstrated that cells could acquire exogenous mitochondria but not exogenous nuclei, indicating that complete cell fusion is unlikely. However, these findings do not definitively rule out the possibility of cell fusion followed by selective loss of the donor cell nuclei. Mitochondria may use TNT to exchange damaged mtDNA for incomplete fusion between two cells under oxidative stress (Guo et al., 2023). Further investigation is needed to determine whether AD cells undergo mitochondrial transfer through complete or partial fusion (Spees et al., 2006).

### 6.4 Endocytosis

Endocytosis is a fundamental cellular process that facilitates active transport across the plasma membrane into cells (Wei et al., 2018). Endocytosed mitochondria are typically found in close proximity to the endogenous mitochondrial network, which is usually located apical to the contractile apparatus (Cowan et al., 2017). Recent reports have suggested that the clathrin-mediated endocytic pathway is one of the main mechanisms of AD (Treusch et al., 2011). However, HepG2 cells can engulf mitochondria via cellular extensions, but clathrin-mediated endocytosis, caveolae-dependent endocytosis, lipid rafts, and other endocytosis mechanisms do not involve interactions with cellular extensions (Kesner et al., 2016). Inhibitors of macrophage phagocytosis may block mitochondrial transfer (Jackson et al., 2016). Nevertheless, understanding regarding how endocytosis specifically regulates mitochondrial transcellular transfer in the context of AD remains limited.

In summary, several hypotheses have been proposed to explain the specific mechanisms underlying mitochondrial transfer (Table 4). Of note, these mechanisms may not be entirely independent from one another. For example, the gap junction protein Cx43 is an important regulator of both TNT formation and function. Moreover, gap junctions and TNTs share some compositional similarities, but they serve distinct functions due to differences in the range of connectivity and the size of molecules they transport. Gap junctions facilitate short-range intercellular interactions, allowing for the transfer of molecules up to 1.2 kDa (Ariazi et al., 2017), whereas TNTs mediate long-range intercellular interactions, allowing greater material transfer. Mitochondrial transfer can occur through either partial or complete cell fusion, often facilitated by TNT formation. Diaphragms are present at TNT-cell junctions, requiring transported substances to pass through this membrane to enter target cells. Additionally, this process may involve cellular endocytosis (Bittins and Wang, 2017). Whether the combination of these communication mechanisms such as TNTs and gap junctions holds significance remains unclear, and clear research evidence or conclusions regarding potential differences in the efficiency and efficacy of different transfer modes is lacking. Therefore, further research is necessary to comprehensively understand the interconnections between these mechanisms and their respective contributions to mitochondrial transfer.

**Table 4:** Various mechanisms involved in mitochondrial transfer.

	Directionality	Secreted cell types	Transferred component	Triggers	Formation	Essential requirements
TNTs	Uni- and bi-directionally (Chakraborty et al., 2023), mainly unidirectional from healthy to damaged cells	Epithelial, fibroblastic, immune, neuronal and glial cells	Mitochondria or fragmented mitochondria	Various risk factors inducing mitochondrial damage	Formed between two connecting cells	Actin polymerization (Zampieri et al., 2021)
EVs	Uni- and bi-directionally (Takenaga et al., 2021)	Almost all cell types, as observed in astrocytes, neurons and mesenchymal stem cells	Mitochondria and mtDNA, EVs are the most likely way to transfer intact mitochondrial particles (Torralba et al., 2016)	Oxygen glucose deprivation	Formed between two isolated cells	–
Gap junction channels (Jain et al., 2023)	Unidirectional	Between neurons, astrocytes, oligodendrocytes, microglia and ependymal cells as well as between different cell types	Mitochondrial or fragmented mitochondria	the factors which can rise the concentrations [Ca^2+^] and [H^+^]	Formed between two connecting cells	Functional protein Cx43
Endocytosis	–	In astrocytes, neurons (Hayakawa et al., 2016)	Free mitochondria or fragmented mitochondria	TNF treatment, starvation treatment (Ren et al., 2019)	–	Dynamic actin filaments
Mitochondrial extrusion	Unidirectional, through cytoplasmic vacuoles originating from the plasma membrane to the extracellular spaces	–	Free mitochondria or fragmented mitochondria	TNF-α, a lot of ROS, or LPS-induced (Unuma et al., 2015)	–	Intact actin and tubulin polymerization
Cell fusion	–	mature stem cells and embryonic stem cells can fuse with neurons	intact mitochondria, fragmented or elongated mitochondria	injury and inflammation (Nygren et al., 2008)	formed between two isolated cells (Wada et al., 2017)	requires an intact mitochondrial inner membrane potential but does not rely on the cytoskeleton (Mattenberger et al., 2003)

Cx43, connexin 43; EV, extracellular vesicle; GJs, gap junctions; LPS, lipopolysaccharide; MIRO, mitochondrial Rho small GTPase; mtDNA, mitochondrial DNA; TNTs, tunneling nanotubes; TNF-α, tumor necrosis factor-α.

## 7 Conclusion

Mitochondrial transfer represents a mode of intracellular and intercellular communication that is widespread among organisms. A considerable body of evidence suggests that movement of mitochondria, both between cells and within individual cells, is a phenomenon observed in mammalian cells both in laboratory settings and in living organisms, occurring under various physiological and pathological conditions. These findings have significantly expanded understanding of mitochondrial transfer. Intracellular and intracellular mitochondrial transfer have different triggering factors, and both contribute to AD pathogenesis. Nevertheless, another connection is also present. Intercellular mitochondrial transfer, a continuation of intracellular mitochondrial transport increases the mtDNA content of the recipient cells and restores their capacity for respiration and survival (Liu et al., 2022).

Dendrites and an axon form a highly polarized structure along the direction of impulse propagation in neurons, which is the basis of intracellular mitochondrial movement (Hirokawa et al., 2010). The intracellular motion of mitochondria is bidirectional, and the direction changes frequently. These movements are regulated by a range of different signals and mechanisms that determine the movement of mitochondria in neurons. Intracellular transport is essential for neuronal development, function, and survival. This intercellular exchange of mitochondria can provide an exogenous mitochondrial source, playing a dynamic role in cellular and tissue responses to CNS injuries. It appears to be essential for the maintenance and restoration of homeostasis within the organism (Wei et al., 2018). Nonetheless, several intricate mechanisms remain to be elucidated. These include the precise processes governing the fusion of TNTs with the target cell membrane, the reception of signals by surrounding cells, the release of EVs, the endocytosis of EVs by target cells, the role of gap junctions and the cytoskeleton in TNTs and EVs, the accurate regulation of mitochondrial transfer mediated by microtubules and microfilaments, and the pathways by which mitochondria are internalized after their release from donor cells and subsequent contact with recipient cells. These areas require further investigation.

Notably, mitochondrial transfer in AD is a bidirectional process, with some transfers proving beneficial for cell survival, while others may potentially exert toxicity on the recipient cells. Despite the growing body of research concerning mitochondrial transfer in AD, many questions remain unanswered. For example, the frequency of this phenomenon *in vivo* remains uncertain, and there is a need to discern whether the exchange of mitochondria observed *in vitro* is merely an artifact of cell culture conditions. Additionally, understanding the degree of cellular damage required to initiate intercellular mitochondrial transfer is crucial. Furthermore, the impact of mitochondrial transfer or transplantation on mitochondrial homeostasis within recipient cells has yet to be comprehensively described in the existing literature. Devoting increased attention to evaluating both the quantity and quality of transferred mitochondria and their subsequent effects on mitochondrial homeostasis during transplantation is imperative. Addressing these unresolved issues necessitates further dedicated research efforts.

Mitochondrial dysfunction stands as a pivotal contributor to AD pathogenesis. Consequently, intracellular and intercellular mitochondrial transfer may be an effective target for the treatment of AD. Restoring mitochondrial function and preserving damaged mitochondria play crucial roles in treating brain injury. Facilitating intercellular mitochondrial transfer through mechanisms such as accelerating neuronal release or enhancing astrocytic phagocytosis holds the potential to be a valuable therapeutic strategy for future AD treatments.
